# The Influence of Ethnicity on Survival from Malignant Primary Brain Tumours in England: A Population-Based Cohort Study

**DOI:** 10.3390/cancers15051464

**Published:** 2023-02-25

**Authors:** Hiba A. Wanis, Henrik Møller, Keyoumars Ashkan, Elizabeth A. Davies

**Affiliations:** 1 Cancer Epidemiology and Cancer Services Research, Centre for Cancer, Society & Public Health, Bermondsey Wing, King’s College London, 3rd Floor, Guy’s Hospital, Great Maze Pond, London SE1 9RT, UK; 2 National Disease Registration Service, NHS England, Leeds LS2 7UE, UK; 3 Danish Centre for Health Services Research, Aalborg University, 9220 Aalborg, Denmark; 4 Department of Neurosurgery, King’s College Hospital NHS Trust, Denmark Hill, London SE5 9RS, UK

**Keywords:** brain tumours, overall survival, ethnicity, health inequalities, cancer registry

## Abstract

**Simple Summary:**

Previous reports using broad ethnic group classifications have suggested that patient outcomes may vary. This study examined survival differences in malignant primary brain tumours of various morphologies between well-recorded and detailed ethnic groups for the whole of England. An ethnic difference in brain tumour survival was found with patients of an Indian background, Any Other White, Other Ethnic Group, and Unknown/Not Stated Ethnicity Groups having better one-year survival than the White British Group, following adjustment for known prognostic factors. By investigating the ethnic variations associated with better brain tumour survival, we may begin to better understand any ethnic inequalities that exist and possibly identify subgroups of patients that could benefit from personalised medicine.

**Abstract:**

Background: In recent years, the completeness of ethnicity data in the English cancer registration data has greatly improved. Using these data, this study aims to estimate the influence of ethnicity on survival from primary malignant brain tumours. Methods: Demographic and clinical data on adult patients diagnosed with malignant primary brain tumour from 2012 to 2017 were obtained (*n* = 24,319). Univariate and multivariate Cox proportional hazards regression analyses were used to estimate hazard ratios (HR) for the survival of the ethnic groups up to one year following diagnosis. Logistic regressions were then used to estimate odds ratios (OR) for different ethnic groups of (1) being diagnosed with pathologically confirmed glioblastoma, (2) being diagnosed through a hospital stay that included an emergency admission, and (3) receiving optimal treatment. Results: After an adjustment for known prognostic factors and factors potentially affecting access to healthcare, patients with an Indian background (HR 0.84, 95% CI 0.72–0.98), Any Other White (HR 0.83, 95% CI 0.76–0.91), Other Ethnic Group (HR 0.70, 95% CI 0.62–0.79), and Unknown/Not Stated Ethnicity (HR 0.81, 95% CI 0.75–0.88) had better one-year survivals than the White British Group. Individuals with Unknown ethnicity are less likely be diagnosed with glioblastoma (OR 0.70, 95% CI 0.58–0.84) and less likely to be diagnosed through a hospital stay that included an emergency admission (OR 0.61, 95% CI 0.53–0.69). Conclusion: The demonstrated ethnic variations associated with better brain tumour survival suggests the need to identify risk or protective factors that may underlie these differences in patient outcomes.

## 1. Background

Each year, over 5000 new cases of primary brain tumours are diagnosed in the United Kingdom (UK) [1]. In particular England has become both more multicultural in recent decades [2] and seen a steady increase in the incidence of malignant primary brain tumours [3]. One study considering broad ethnic groups found higher incidence rates of 4.8 per 100,000 population from 2001 to 2007 for people from White ethnic group compared to those from South Asian, Black, and Chinese ethnic groups (respective rates of 3.1, 2.8, and 2.7 per 100,000 population) [4].

English population-based studies have reported ethnic differences in the incidence of most cancers with individuals from non-White groups generally having a lower cancer risk than the White Group [5,6]. Survival for the four common cancers has been widely reported [6], but not examined in detail for brain tumours using the well-defined ethnicity information now available. A small study of high-grade gliomas in South-East England 2000–2009 [7], reported that patients of White and Not Known ethnicities had the worse survival for all tumour groups after adjusting for sex, age, morphology, socio-economic deprivation, and co-morbidity.

The improved and detailed National Health Service (NHS) data on ethnicity captured by the National Disease Registration Service, which is part of NHS England, provided an opportunity to explore the impact of ethnicity on brain tumour survival. This resulted from major efforts across the NHS to increase self-reporting of this variable. With data collected on over 300,000 cancer cases in England each year, this is also the first English study to consider the more detailed classifications for malignant primary brain tumours including all gliomas, primary central nervous system lymphoma (PCNSL), as well as unclassified malignant brain neoplasms. It aims to examine the possible effect of ethnicity on the route or pathway taken to diagnosis and of receiving optimal treatment. A better understanding of any ethnic inequalities in brain cancer could potentially lead to improved treatment or services for these patients.

## 2. Data and Methods

### 2.1. Study Population

Data on all adult patients diagnosed with a malignant primary brain tumour during 2012–2017, who are resident in England and registered with a general practitioner (GP), were extracted from the English cancer registration data.

### 2.2. Selection of Cases

Cases for this study were identified using the International Classification of Diseases [version 10] (ICD-10) tumour site C71. For those with PCNSL, ICD-10 code site was used along with the morphology codes for lymphoma. Other inclusion criteria were cases having a complete tumour registration and known sex. The brain tumour morphological subtypes considered in this study were based on the 2016 WHO Classification of Tumours of the Central Nervous System [8]. WHO updated this classification in 2021; however, the changes have minimal effect on the analysis of this data. Due to sample sizes, histological tumour subtypes were grouped as follows: glioblastoma, anaplastic astrocytoma, astrocytoma NOS, oligodendroglioma, PCNSL, malignant glioma, and unclassified malignant. Data on all tumours were extracted from the English cancer registration irrespective of their pathological confirmation—gliomas without a specified classification or as unclassified malignant neoplasms were included. In addition, glioblastomas with a pathological confirmation were included but tumours of benign, uncertain, and metastatic nature were not included. Molecular data are not available for this study cohort.

Inpatient hospital episodes statistics (HES) data were linked to the cancer registration data from 2012. These records include ethnicity data that are almost always self-reported upon admission to NHS hospitals. The categories of ethnicities were as follows: White British, Bangladeshi, Indian, Pakistani, Chinese, Black African, Black Caribbean, and Unknown/Not Stated, and due to small numbers in these groups—White Irish and Any Other White were grouped together as Any Other White, and Mixed Ethnic Groups and Any Other Ethnic Group were grouped as Other Ethnic Group.

Socio-economic deprivation was measured using the income domain of the index of multiple deprivation (IMD) 2015, divided into quintiles across England and Wales, and assigned to cases using postcode of residence at diagnosis. Charlson co-morbidity score was based on conditions occurring within one year of the cancer diagnosis date. The conditions were weighted according to their severity and scores were grouped as 0 (where none were recorded), 1, and 2 or more. Route to Diagnosis is defined as the sequence of interactions between the patient and the NHS, leading to a cancer diagnosis [9]. This is identified using an algorithm linking various sources based on the setting of diagnosis, and the pathway and referral route into secondary care.

Information on surgical resections, chemotherapy, and radiotherapy treatments received within the first 18 months following diagnosis were also extracted. Treatment options were categorised to reflect clinical practice as: radiotherapy only, chemotherapy only, surgical resection only, all three treatments given as surgical resection followed by radiotherapy and chemotherapy (optimal treatment), radiotherapy plus chemotherapy, surgical resection plus radiotherapy, surgical resection plus chemotherapy, and no treatment. Surgical resections did not include cases with biopsies only.

### 2.3. Data Analysis

**Figure 1:**
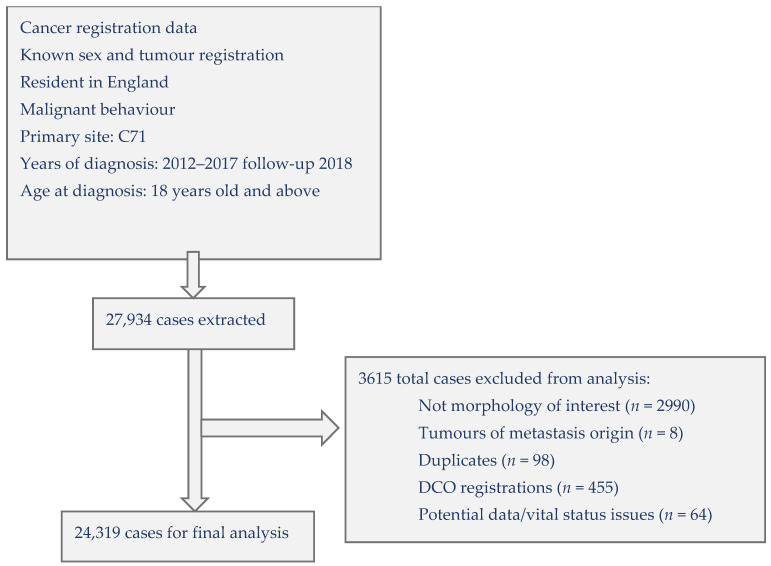
Selection of cases for analysing the influence of ethnicity on malignant primary brain tumour survival in England, 2012–2017 with follow-up to 2018 (DCO = Death Certificate Only).

We first extracted 27,934 records, cleaning the dataset to exclude duplicated cases, those without the required brain tumour morphology, with unknown vital status, or who were registered by death certificate only (DCO) (Figure 1). Survival time was calculated from the date of diagnosis until date of death with a survival period of up to one year. To retain 145 patients who died on their date of diagnosis, we added half a day to their survival time. The final study population included 24,319 cases.

Initially, we examined the distribution of patients by demographic factors (age, sex, area of residence, and socio-economic status), co-morbidity, tumour morphology, route to diagnosis, and treatment factors. Univariate and multivariate Cox proportional hazards regressions were then used to estimate hazard ratios (HR) and their 95% confidence intervals (95% CI) for the survival of each ethnic group up to one year following diagnosis. The follow-up period ended on 31 December 2018. χ^2^ Tests estimated the p-values for trend and heterogeneity, excluding unknown categories. We then carried out a sensitivity analysis in which each variable was adjusted to identify how much variation it contributed to the model, and as a result we finally focused the analysis on age, sex, co-morbidity, socio-economic deprivation, tumour morphology, route to diagnosis, and treatment received. Due to the high fatality of malignant primary brain tumours, cancer-specific survival was not studied, as this is similar to overall survival.

Logistic regression was used to generate odds ratios (OR) (and their 95% CI) for each ethnic group of (1) being diagnosed with pathologically confirmed glioblastoma, (2) being diagnosed during a hospital stay that included an emergency admission, and (3) receiving optimal treatment (surgical resection followed by radiotherapy and chemotherapy). ORs were adjusted for age, sex, socio-economic deprivation, co-morbidity, morphology, route to diagnosis (patient’s pathway to diagnosis), and treatment. All analyses were performed using Stata Software, version 16 (StataCorp, TX, USA).

### 2.4. Ethical Approval

Data for this study were collected and analysed under the National Disease Registries Directions 2021, made in accordance with sections 254(1) and 254(6) of the 2012 Health and Social Care Act. Further ethical approval for this study was not required per the definition of research according to the UK Policy Framework for Health and Social Care Research.

## 3. Results

**Figure 2:**
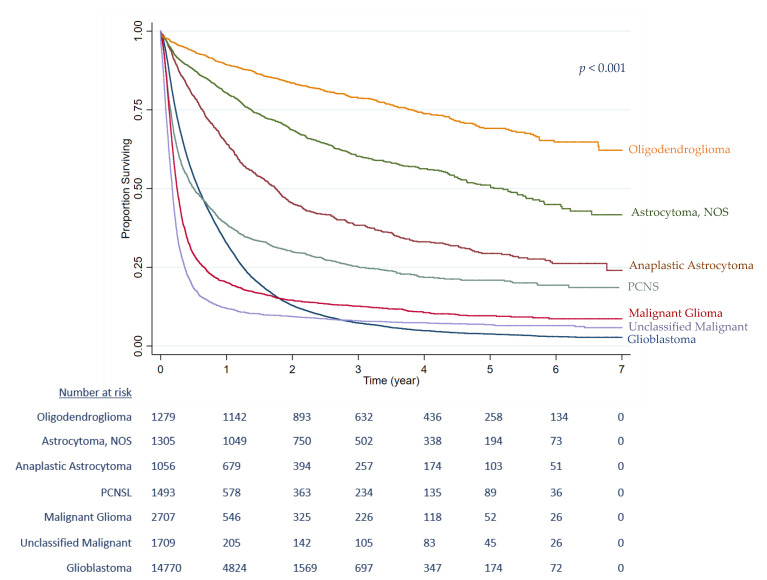
Kaplan-Meier survival curves by brain tumour morphology, England 2012–2017.

Data from 24,319 patients with a malignant primary brain tumour diagnosed between 2012 and 2017 in England were included. Table 1 displays the distribution of patient, tumour and clinical characteristics, and univariate and mutually adjusted HRs. Brain tumour diagnosis increased with age, peaking at 65–74 years with most patients being men (58.0% *n* = 14,094). In absolute numbers, it was more frequent in people living in Southeast England, an area that is highly populated and more ethnically diverse. Overall, the most aggressive morphology, glioblastoma, was the most common type (60.7% *n* = 14,768). The Kaplan–Meier analysis for brain tumour morphology (Figure 2) demonstrates glioblastoma as having a very high mortality, followed by malignant glioma (7.0% of all cases, *n* = 1709) and unclassified malignant tumours (11.1% of all cases, *n* = 2707) (log-rank test, *p* < 0.001). Over one half of cases (53.2% *n* = 12,926) were diagnosed during a hospital stay that included an emergency admission, with most patients receiving either the optimal treatment (23.0% *n* = 5585), or no treatment (34.9% *n* = 8483). In the univariate analysis, each of the covariates was correlated with survival. The effects of age, sex, and co-morbidity were attenuated in the mutually adjusted analyses.

Almost all patients (95.6%) were recorded as having a known ethnicity. The most common ethnic group representing 85.5% (*n* = 20,795) of the patients was the White British Group, followed by 4.2% (*n* = 1018) from the Any Other White Group and 2.8% (*n* = 674) from Other Ethnic Group. The more specific ethnic groups were less common with 1.3% (*n* = 321) of patients defining themselves as Indian, 0.8% (*n* = 186) as Pakistani, and less than 0.4% as Bangladeshi (*n* = 30), Chinese (*n* = 37), Black African (*n* = 84), and Black Caribbean (*n* = 94) (Table 2). The univariate model for ethnicity showed a survival difference and the mutually adjusted model demonstrated that patients with Other Ethnic Group and Unknown/Not Stated Ethnicity had a 18% and 23% decreased risk of death from any cause, respectively, compared to the White British Group.

In a sensitivity analysis, the association of survival with age seemed to disappear in most non-white ethnic groups. This could be explained by the younger age of these groups leading to a lower median age at diagnosis than for the White British population (Table 2). The effect on survival in the Unknown/Not Stated Group was less sensitive to statistical adjustment by age, as the median age was older than for the White British Group. After fully adjusting for age, sex, co-morbidity, socio-economic deprivation, tumour morphology, route to diagnosis and treatment received, patients from the Indian Group (HR 0.84, 95% CI 0.72–0.98), Any Other White (HR 0.83, 95% CI 0.76–0.91), Other Ethnic Group (HR 0.70, 95% CI 0.62–0.79) and Unknown/Not Stated Ethnicity (HR 0.81, 95% CI 0.75–0.88), had better one-year survivals than the White British Group (Table 3). There was no difference between the White British Group and the remaining Bangladeshi, Pakistani, Chinese, Black Caribbean, and Black African Ethnic minority groups.

The ethnic difference in survival was further explored by investigating whether there was any interaction between ethnicity and glioblastoma diagnosis, route or pathway to diagnosis, and optimal treatment received (Table 4). The Any Other White Group were more likely to be diagnosed through a hospital stay that included an emergency admission (OR 1.16, 95% CI 1.02–1.33). The Other Ethnic Group were nearly a third more likely to receive the diagnosis of glioblastoma (OR 1.28, 95% CI 1.04–1.56) than the White British Group. However, individuals with Unknown/Not Stated Ethnicity had the most favourable prognosis and were less likely to be diagnosed with a glioblastoma (OR 0.70, 95% CI 0.58–0.84), less likely to be diagnosed through a hospital stay that included an emergency admission (OR 0.61, 95% CI 0.53–0.69), and more likely to receive the optimal treatment option for their other-than-glioblastoma diagnosis (OR 0.39, 95% CI 0.31–0.49).

## 4. Discussion

### 4.1. Main Findings

This study of 24,319 people residing in England and diagnosed with a brain tumour between 2012 and 2017 shows better one-year survival for patients from Indian, Any Other White, Other Ethnic Groups, and Unknown/Not Stated Ethnic Groups than for the White British Group (HR 0.84 (95% CI 0.72–0.98), HR 0.83 (95% CI 0.76–0.91), HR 0.70 (95% CI 0.62–0.79), and HR 0.81 (95% CI 0.75–0.88), respectively). The survival analysis was adjusted for age, sex, co-morbidity, socio-economic deprivation, tumour morphology, route to diagnosis, and treatment received. Individuals with Unknown/Not Stated Ethnicity had the best prognoses and as a group were less likely be diagnosed with glioblastoma or to be diagnosed through a hospital stay, including an emergency admission.

### 4.2. Comparisons to Other Findings

In comparison to the smaller study by Ratneswaren et al. (2014), which was limited to high-grade glioma patients living in South East England [7], our current study was able to incorporate additional factors that may influence the impact of ethnicity on survival. In this larger national dataset, the heterogenous ethnicities were categorised into better defined groups for a precise analysis. We also found the Indian and Other Ethnic Group had a better survival than the White British Group. However, we identified the Unknown/Not Stated Ethnic Group having a better one-year survival than the White British Group, in contrast to the reverse finding in the earlier study. This could be due to a higher proportion of unknown ethnicity data, which was 21.7% compared to only 4.4% in the current study.

US population-based studies have also reported racial and ethnic variations in brain tumour incidence and survival. Most have shown that Caucasian people have poorer survival outcomes compared to Black/African Americans and Asian/Pacific Islander Americans [10,11,12,13,14,15]. Other work, however, has reported that African Americans have an increased risk of death from malignant brain tumour compared to Caucasians and other race and ethnicities [16], which was explained further by an interaction between race and surgery type.

### 4.3. Interpretations and Implications

In this study, we have demonstrated that the White British Ethnic Group has a poorer survival compared to other ethnic groups. An English paper by Maile et al. (2016) has reported incidence data broadly similar to the US finding that patients from White Ethnic Groups were significantly more likely to develop glioblastoma than other racial/ethnic groups [4,17]. They did not evaluate survival; however, their results could help explain the association between White British ethnicity and a higher risk of mortality from high-grade glioma.

Increasing age is known to be a poor prognostic factor for patients with malignant brain tumour [17]. The demography of ethnic minorities in England reflects the fact that people from these groups are younger and congregate in major cities, such as London, compared to other areas of England. A cohort study from the US also identified that patients of Hispanic background were diagnosed at a younger age compared to non-Hispanic Whites and had an improved overall survival [18]. A report by The King’s Fund suggested that, overall, people from ethnic minorities have poorer access to UK healthcare compared to the White British Groups [19], and this could correlate with fewer individuals from these being registered with the NHS. As a result, the probability of White people being diagnosed with a glioma could be increased due to their greater use of diagnostic tools, even from a young age, and therefore, they have a greater risk of ionising radiation exposure [20]. Since our finding of better survival for patients from Any Other White and Unknown/Not Stated Ethnic Group is new, it needs further exploration. One explanation could be that these individuals travel to their countries of origin for better healthcare and social support following their diagnosis, which could mean that their deaths abroad were not formally registered in the English system [21].

Brain tumours are considered difficult to diagnose [22], as these cancers tend to (1) involve 3 or more GP visits before diagnosis [23,24] and (2) are likely to present as an emergency [9,25]; both could lead to poorer outcomes [9]. The potential impact of family support on outcome might also differ between ethnic groups. People from minority ethnic groups, particularly those from Asian backgrounds, are more likely to be surrounded by extended families compared to the nuclear family structure, typical of the White British Group; it is possible that extended families may be more likely to recognise subtle signs of a brain tumour, including neuro-cognitive changes, as well as possible recurrences and encourage earlier diagnosis.

The current standard therapy for gliomas consists of surgical resection followed by adjuvant chemotherapy and radiation, prolonging median overall survival to 15 months for glioblastomas [26,27], and is represented in this study by the optimal treatment option. From our results, we demonstrated that the Unknown/Not Stated Ethnic Group are less likely to receive optimal treatment, but that could be due to the lower chance of being diagnosed with a glioblastoma.

Studies investigating possible factors explaining brain tumour occurrence have identified genes that could be associated with glioma development and tumours carrying the worst prognosis [28,29]. The presence of such genes and their specific alterations could perhaps explain the differences in prognosis by ethnicity. Epigenetic age acceleration, which is the difference between age predicted by DNA methylation and chronological age, has been linked with many cancers [30]. A recent study by Crimmins et al. (2021), which examined epigenetic clocks to evaluate a linkage with race/ethnicity, found that the majority of clocks indicated slower epigenetic ageing among Hispanic and African American individuals compared to White individuals [31]. The reports of differing incidence and survival by racial/ethnic groups, make it important to explore possible genetic alterations and variation in signalling pathways to identify and compare polymorphisms between ethnic minority and White individuals [13]. For example, one study found a 42% reduction of risk of glioma in patients with a history of diabetes [32], and a recent meta-analysis confirmed this inverse relationship where elevated blood sugar, or a previous history of diabetes, are inversely associated with risk of glioma [33]. In England, people of Black (African and Caribbean) and South Asian (Bangladeshi, Indian, and Pakistani) backgrounds are at higher risk of developing type 2 diabetes from a younger age compared to those of a White background [34], and this could possibly be associated with the decreased glioma mortality observed here.

The better survival for Indian individuals after adjusting for other factors was of particular interest and may suggest that there are other more specific influencing factors. As there were no significant differences between Indian and White British Groups in terms of patient characteristics, tumour morphology, and route or pathway to diagnosis, further explanation is needed to justify the difference when treatment received is added to the Cox proportional regression model. Due to curcumin’s anti-tumoural effects on glioma cells in preclinical in vitro and in vivo [35], we speculate that an Indian diet that usually includes curcumin might play a role in their prolonged brain tumour survival, perhaps linked to a better response to treatment. A recent study in India evaluated the molecular biomarkers of brain tumours in Indian patients [36] and reported a high prevalence of isocitrate dehydrogenase 1 (IDH1) mutation in astrocytoma and glioblastoma in these patients [37]. The presence of IDH1 mutation correlates with a survival benefit and is more common among glioblastomas progressing from a lower grade glioma compared with 5–10% of de novo glioblastomas [38,39]. Consequently, the association between individuals of an Indian background and improved survival could be related in some way to this mutation, in addition to, or perhaps independent of, the therapeutic potential of curcumin. Obtaining detailed information on molecular, genetics, and lifestyle or environmental factors could enable us to further compare outcomes with other populations.

### 4.4. Strengths and Limitations

The extended period of time covered by this study, along with the greatly improved cancer registration data in recent years for England, have meant that more detailed ethnic groups can be analysed. The previously reported NCIN incidence data for the years 2002 to 2006, and other related studies, found a quarter of cancer patients had unknown (i.e., missing) ethnicity information [4,6,7]. While cancer registration acknowledges that ethnicity data may not be self-reported and may possibly have been derived from already held information, the very large increase in completeness means we can be confident in these analyses. In these new data, only 4% of patients had unknown/not stated ethnicity information and were analysed separately to get a better understanding of this individual group. Our study also considered the importance of analysing more defined ethnic groups, as significant heterogeneity of risk for many cancers can be seen particularly among those from Black and South Asian Groups [4]. Another strength of this study is the adjustment for many prognostic variables that could vary by ethnicity and prognosis. However, other and unknown factors could still be relevant including patient’s performance status, tumour location within the brain, extent of tumour excision, and more interestingly, molecular biomarkers and genetic information. Our study had some limitations. Reporting patterns of incidence for the histological subtypes by ethnicity would have strengthened the study; however, this was restricted by the small number of patients in each subgroup. In addition, limited numbers of patients in the mixed ethnic groups and those from a White Irish background meant they had to be combined within Any Other Ethnic and Any Other White Groups respectively. Additionally, cancer registration did not collate information that could possibly influence survival, such as recurrences, glioblastoma progressing from a low-grade glioma, or biopsy-only—and hence, we observed a high proportion of patients (34.9%) who had no surgical resection but could possibly just had a diagnostic biopsy.

## 5. Conclusions

To obtain a better understanding of potential ethnic differences in malignant primary brain tumour survival, we carried out a detailed evaluation of factors, including age at diagnosis, sex, co-morbidity, socio-economic deprivation, histologic tumour subtype (which is correlated with tumour grade), route or pathway to diagnosis, and treatment options that might affect prognosis. After controlling for these variables, we found that patients from Indian, Any Other White, Other Ethnic Groups, and Unknown/Not Stated Ethnic Groups, had better one-year survival compared to the White British Group. To determine whether biological, behavioural, or clinical factors are driving these survival differences, more data on patients’ clinicopathological characteristics are therefore needed. This will help us better understand any ethnic inequalities in brain cancer and identify improvements to the health service for specific groups.

## Figures and Tables

**Table 1:** Distribution of covariates in survival analysis of malignant primary brain tumours patients diagnosed in England, 2012–2017 with follow-up to 2018. Univariate and mutually adjusted Cox proportional hazards regression analyses to estimate hazard ratios.

	Cases	%	Deaths	Univariate		Mutually Adjusted	
				HR (95% CI)		HR (95% CI)	
**Age at diagnosis**							
<=34	1549	6.4	538	1.00		1.00	
35–44	1662	6.8	802	1.44	(1.22, 1.70)	1.54	(1.30, 1.82)
45–54	3158	13.0	2278	2.84	(2.46, 3.26)	2.64	(2.29, 3.04)
55–64	4993	20.5	4285	4.89	(4.28, 5.59)	3.95	(3.45, 4.53)
65–74	6667	27.4	6184	8.11	(7.11, 9.26)	4.95	(4.32, 5.67)
75–84	4738	19.5	4635	15.69	(13.75, 17.91)	5.31	(4.63, 6.09)
>=85	1552	6.4	1545	26.21	(22.81, 30.12)	6.37	(5.52, 7.36)
*Trend*				*χ2 (1) = 7273.09; p < 0.001*		*χ2 (1) = 1279.79; p < 0.001*	
**Sex**							
Male	14,094	58.0	11,828	1.00		1.00	
Female	10,225	42.1	8439	1.07	(1.03, 1.10)	0.96	(0.92, 0.99)
*Heterogeneity*				*χ^2^ (1) = 15.63; p < 0.001*		*χ^2^ (1) = 7.60; p < 0.001*	
**Area of residence**							
East Midlands	2268	9.3	1913	1.24	(1.16, 1.34)	1.24	(1.15, 1.34)
East of England	2837	11.7	2404	1.41	(1.31, 1.51)	1.18	(1.10, 1.27)
London	2573	10.6	1970	1.00		1.00	
North East	1414	5.8	1212	1.35	(1.24, 1.47)	1.22	(1.12, 1.33)
North West	3318	13.6	2747	1.21	(1.13, 1.30)	1.36	(1.27, 1.46)
South East	4094	16.8	3415	1.27	(1.19, 1.36)	1.10	(1.03, 1.18)
South West	2895	11.9	2483	1.38	(1.29, 1.48)	1.15	(1.07, 1.24)
West Midlands	2409	9.9	2041	1.31	(1.22, 1.41)	1.34	(1.24, 1.44)
Yorkshire and The Humber	2511	10.3	2082	1.31	(1.22, 1.41)	1.17	(1.09, 1.26)
*Heterogeneity*				*χ^2^ (8) = 126.13; p < 0.001*		*χ^2^ (8) = 110.88; p < 0.001*	
**Socio-economic deprivation**							
1 least deprived	5615	23.1	4705	1.00		1.00	
2	5607	23.1	4724	1.07	(1.03, 1.13)	1.04	(0.99, 1.09)
3	4985	20.5	4169	1.09	(1.04, 1.14)	1.06	(1.01, 1.11)
4	4308	17.7	3548	1.09	(1.03, 1.14)	1.08	(1.03, 1.14)
5 most deprived	3804	15.6	3121	1.09	(1.04, 1.15)	1.17	(1.11, 1.24)
*Trend*				*χ^2^ (1) = 11.53; p < 0.001*		*χ^2^ (1) = 31.88; p < 0.001*	
**Charlson co-morbidity score**							
0	20,400	83.9	16,660	1.00		1.00	
1	2108	8.7	1901	1.60	(1.52, 1.69)	1.07	(1.01, 1.12)
2+	1811	7.5	1706	2.19	(2.07, 2.31)	1.11	(1.05, 1.17)
*Trend*				*χ^2^ (1) = 1031.64; p < 0.001*		*χ^2^ (1) = 16.55; p < 0.001*	
**Morphology**							
Glioblastoma	14,768	60.7	13,619	1.00		1.00	
Astrocytoma, Anaplastic	1058	4.4	675	0.40	(0.36, 0.44)	0.58	(0.53, 0.65)
Astrocytoma, NOS	1306	5.4	557	0.20	(0.18, 0.23)	0.20	(0.18, 0.23)
Oligodendroglioma	1279	5.3	321	0.11	(0.09, 0.13)	0.12	(0.10, 0.15)
PCNSL	1492	6.1	1124	0.97	(0.91, 1.04)	0.67	(0.62, 0.72)
Malignant Glioma	2707	11.1	2386	1.68	(1.60, 1.76)	0.67	(0.63, 0.70)
Unclassified Malignant	1709	7.0	1585	2.48	(2.34, 2.61)	0.79	(0.75, 0.84)
*Heterogeneity*				*χ^2^ (6) = 3293.59; p < 0.001*		*χ^2^ (6) = 1345.58; p < 0.001*	
**Treatment**							
Radiotherapy only	2176	9.0	1964	1.00		1.00	
Chemotherapy only	916	3.8	642	0.65	(0.59, 0.72)	0.80	(0.72, 0.90)
Surgical resection only	2317	9.5	1563	0.86	(0.80, 0.93)	1.56	(1.45, 1.68)
Surgical resection + Radiotherapy + Chemotherapy	5585	23.0	4200	0.24	(0.22, 0.26)	0.23	(0.21, 0.25)
Radiotherapy + Chemotherapy	1994	8.2	1588	0.40	(0.37, 0.44)	0.44	(0.41, 0.48)
Surgical resection + Radiotherapy	2525	10.4	2127	0.63	(0.58, 0.67)	0.57	(0.53, 0.61)
Surgical resection + Chemotherapy	323	1.3	227	0.46	(0.39, 0.54)	0.72	(0.60, 0.85)
No treatment	8483	34.9	7956	2.51	(2.38, 2.65)	2.47	(2.33, 2.62)
*Heterogeneity*				*χ^2^ (7) = 9705.48; p < 0.001*		*χ^2^ (7) = 6350.45; p < 0.001*	
**Route to Diagnosis**							
Emergency presentation	12,926	53.2	11,622	1.00		1.00	
GP referral	4833	19.9	3668	0.55	(0.52, 0.57)	0.73	(0.70, 0.76)
Inpatient elective	743	3.1	595	0.49	(0.44, 0.54)	0.87	(0.78, 0.96)
Other outpatient	4791	19.7	3569	0.46	(0.44, 0.48)	0.80	(0.77, 0.84)
Two Week Wait (TWW) Urgent referral	428	1.8	355	0.62	(0.55, 0.70)	0.82	(0.72, 0.93)
Unknown	598	2.5	458	0.69	(0.62, 0.77)	0.60	(0.54, 0.67)
*Heterogeneity (excluding unknown)*				*χ^2^ (4) = 1646.89; p < 0.001*		*χ^2^ (4) = 230.11; p < 0.001*	

**Table 2:** Survival analysis of 24,319 patients diagnosed with malignant primary brain tumour in England, 2012–2017, by ethnicity.

							Univariate		Mutually Adjusted	
Ethnic Group	Number of Patients	%	Number of Deaths	Median Age	Median Survival/Months	(95% CI)	HR (95% CI)		HR (95% CI)	
White British	20,795	85.5	17,653	66.0	17.0	(16.6, 17.5)	1.00	(Ref)	1.00	(Ref)
Bangladeshi	30	0.1	22	61.5	18.0	(8.5, 85.5)	0.98	(0.62, 1.53)	1.19	(0.76, 1.88)
Indian	321	1.3	231	59.0	27.7	(23.4, 36.0)	0.72	(0.62, 0.84)	0.89	(0.76, 1.03)
Pakistani	186	0.8	130	56.0	26.0	(19.1, 34.5)	0.75	(0.61, 0.91)	0.95	(0.77, 1.16)
Chinese	37	0.2	26	57.0	25.3	(11.7, 50.7)	0.85	(0.56, 1.30)	1.16	(0.76, 1.76)
Black African	84	0.4	59	53.5	29.8	(20.8, 47.7)	0.67	(0.50, 0.91)	0.98	(0.72, 1.33)
Black Caribbean	94	0.4	78	60.5	19.6	(13.2, 33.8)	0.85	(0.65, 1.11)	0.86	(0.66, 1.12)
Any Other White	1018	4.2	747	61.0	26.7	(24.0, 31.5)	0.73	(0.67, 0.80)	0.88	(0.81, 0.96)
Other Ethnic Groups	674	2.8	445	53.0	40.5	(36.5, 47.4)	0.53	(0.47, 0.60)	0.77	(0.68, 0.87)
Unknown/Not Stated	1080	4.4	876	68.0	10.2	(9.4, 11.8)	1.25	(1.16, 1.35)	0.82	(0.76, 0.88)
*Heterogeneity (excluding unknown)*							*χ^2^ (8) = 182.58; p < 0.001*		*χ^2^ (8) = 29.78; p < 0.001*	

**Table 3:** Survival analysis of different ethnic groups of patients diagnosed with malignant primary brain tumour in England, 2012–2017.

	Adjusted for Age, Sex			and Socioeconomic Deprivation, Co-morbidity			and Morphology			and Route to Diagnosis			and Treatment Received		
Ethnic Group	HR	(95% CI)	*p*-Value	HR	(95% CI)	*p*-Value	HR	(95% CI)	*p*-Value	HR	(95% CI)	*p*-Value	HR	(95% CI)	*p*-Value
White British	1.00	(Ref)		1.00	(Ref)		1.00	(Ref)		1.00	(Ref)		1.00	(Ref)	
Bangladeshi	1.47	(0.94, 2.30)	0.094	1.24	(0.79, 1.95)	0.341	1.15	(0.74, 1.81)	0.531	1.15	(0.73, 1.80)	0.546	1.05	(0.67, 1.65)	0.824
Indian	0.92	(0.79, 1.07)	0.290	0.89	(0.76, 1.04)	0.142	0.91	(0.78, 1.06)	0.213	0.90	(0.77, 1.05)	0.178	0.84	(0.72, 0.98)	0.025
Pakistani	1.16	(0.95, 1.42)	0.153	1.00	(0.82, 1.23)	0.989	1.04	(0.85, 1.27)	0.707	1.00	(0.82, 1.23)	0.984	0.96	(0.78, 1.17)	0.658
Chinese	1.29	(0.85, 1.96)	0.231	1.24	(0.82, 1.88)	0.315	1.22	(0.80, 1.85)	0.356	1.23	(0.81, 1.86)	0.342	1.09	(0.72, 1.66)	0.690
Black African	1.08	(0.80, 1.46)	0.611	0.98	(0.72, 1.33)	0.888	0.91	(0.67, 1.24)	0.551	0.90	(0.66, 1.22)	0.496	0.87	(0.64, 1.17)	0.355
Black Caribbean	1.02	(0.78, 1.33)	0.868	0.90	(0.69, 1.17)	0.416	0.84	(0.65, 1.10)	0.207	0.82	(0.63, 1.07)	0.153	0.81	(0.62, 1.06)	0.126
Any Other White	0.91	(0.83, 0.99)	0.034	0.90	(0.82, 0.98)	0.014	0.90	(0.83, 0.98)	0.019	0.89	(0.81, 0.97)	0.007	0.83	(0.76, 0.91)	<0.001
Other Ethnic Groups	0.84	(0.74, 0.94)	0.003	0.80	(0.71, 0.90)	<0.001	0.80	(0.71, 0.90)	<0.001	0.79	(0.70, 0.89)	<0.001	0.70	(0.62, 0.79)	<0.001
Unknown/Not Stated	1.14	(1.06, 1.23)	<0.001	1.17	(1.09, 1.26)	<0.001	1.11	(1.03, 1.19)	0.008	1.10	(1.02, 1.18)	0.016	0.81	(0.75, 0.88)	<0.001
*Heterogeneity (excluding unknown)*	*χ^2^ (8) = 20.85; p < 0.001*			*χ^2^ (8) = 23.81; p < 0.001*			*χ^2^ (8) = 23.76; p < 0.001*			*χ^2^ (8) = 25.32; p < 0.001*			*χ^2^ (8) = 56.43; p < 0.001*		

The HR were adjusted for age and sex, and then, they were sequentially adjusted for socio-economic and co-morbidity, morphology, route to diagnosis, and treatment received.

**Table 4:** Odds ratios of malignant primary brain tumour patients in England 2012–2017, by ethnicity, diagnosed with glioblastoma, diagnosed as emergency through hospital stay and receiving optimal treatment. Percentage (%) of patients within each ethnic group.

	Pathologically Confirmed Glioblastoma Diagnosis ^1^				Diagnosed through a Hospital Stay That Included an Emergency Admission ^2^				Optimal Treatment ^3^			
Ethnic Group	% Patients	OR	(95% CI)	*p* Value	% Patients	OR	(95% CI)	*p* Value	% Patients	OR	(95% CI)	*p* Value
White British	56.0	1.00			53.2	1.00			24.8	1.00		
Bangladeshi	56.7	1.77	(0.79, 3.99)	0.168	50.0	0.80	(0.38, 1.69)	0.559	13.3	0.42	(0.13, 1.31)	0.136
Indian	54.1	1.14	(0.86, 1.52)	0.360	53.9	1.19	(0.94, 1.50)	0.148	24.9	0.81	(0.61, 1.08)	0.150
Pakistani	40.6	0.74	(0.51, 1.08)	0.121	59.1	1.34	(0.98, 1.83)	0.068	18.3	0.66	(0.44, 1.00)	0.049
Chinese	48.6	1.22	(0.54, 2.77)	0.638	48.6	0.90	(0.45, 1.79)	0.765	18.9	0.53	(0.22, 1.28)	0.156
Black African	48.7	1.10	(0.63, 1.92)	0.741	54.8	1.18	(0.75, 1.84)	0.474	20.2	0.68	(0.38, 1.23)	0.203
Black Caribbean	51.8	0.96	(0.56, 1.65)	0.893	56.4	1.06	(0.69, 1.63)	0.799	27.7	1.27	(0.75, 2.14)	0.377
Any Other White	54.1	1.14	(0.96, 1.34)	0.124	54.6	1.16	(1.02, 1.33)	0.029	26.6	0.95	(0.81, 1.12)	0.545
Other Ethnic Groups	54.2	1.28	(1.04, 1.56)	0.017	51.0	1.10	(0.94, 1.30)	0.233	31.0	0.99	(0.82, 1.19)	0.911
Unknown/Not Stated	33.6	0.70	(0.58, 0.84)	<0.001	50.1	0.61	(0.53, 0.69)	<0.001	9.4	0.39	(0.31, 0.49)	<0.001

^1^ Adjusted for age, sex, socio-economic deprivation, co-morbidity, route to diagnosis, treatment. ^2^ Adjusted for age, sex, socio-economic deprivation, co-morbidity, morphology, treatment. ^3^ Adjusted for age, sex, socio-economic deprivation, co-morbidity, morphology, route to diagnosis.

## Data Availability

Data may be obtained from a third party and are not publicly available. The data that support the findings of this study are available from NHS England but restrictions apply to the availability of these data, which were used under license for the current study, and so are not publicly available. The authors do not own these data, and therefore are not permitted to share or provide these data other than in scientific communication format.

## References

[1] Cancer Research UK Brain, Other CNS and Intracranial Tumours Statistics. https://www.cancerresearchuk.org/health-professional/cancer-statistics/statistics-by-cancer-type/brain-other-cns-and-intracranial-tumours?_ga=2.172768099.1375741702.1600433248-1594617780.1600433248.

[2] ONS Ethnicity Statistics for England. https://www.ons.gov.uk/peoplepopulationandcommunity/culturalidentity/ethnicity.

[3] Wanis H.A., Møller H., Ashkan K., Davies E.A. (2021). The incidence of major subtypes of primary brain tumors in adults in England 1995–2017. Neuro-Oncology.

[4] Maile E.J., Barnes I., Finlayson A.E., Sayeed S., Ali R. (2016). Nervous System and Intracranial Tumour Incidence by Ethnicity in England, 2001–2007: A Descriptive Epidemiological Study. PLoS ONE.

[5] Delon C., Brown K.F., Payne N.W.S., Kotrotsios Y., Vernon S., Shelton J. (2022). Differences in cancer incidence by broad ethnic group in England, 2013–2017. Br. J. Cancer.

[6] Thomson C., Forman D. (2009). Cancer Incidence and Survival by Major Ethnic Group, England, 2002–2006.

[7] Ratneswaren T., Jack R.M., Tataru D., Davies E.A. (2014). The survival of patients with high grade glioma from different ethnic groups in South East England. J. Neurooncol..

[8] Louis D.N., Perry A., Wesseling P., Brat D.J., Cree I.A., Figarella-Branger D., Hawkins C., Ng H.K., Pfister S.M., Reifenberger G. (2021). The 2021 WHO Classification of Tumors of the Central Nervous System: A summary. Neuro-Oncology.

[9] Elliss-Brookes L., McPhail S., Ives A., Greenslade M., Shelton J., Hiom S., Richards M. (2012). Routes to diagnosis for cancer-determining the patient journey using multiple routine data sets. Br. J. Cancer.

[10] Jemal A., Murray T., Samuels A., Ghafoor A., Ward E., Thun M.J. (2003). Cancer statistics, 2003. CA Cancer J. Clin..

[11] Patel N.P., Lyon K.A., Huang J.H. (2019). The effect of race on the prognosis of the glioblastoma patient: A brief review. Neurol. Res..

[12] Ostrom Q.T., Patil N., Cioffi G., Waite K., Kruchko C., Barnholtz-Sloan J.S. (2020). CBTRUS Statistical Report: Primary Brain and Other Central Nervous System Tumors Diagnosed in the United States in 2013–2017. Neuro-Oncology.

[13] Gabriel A., Batey J., Capogreco J., Kimball D., Walters A., Tubbs R.S., Loukas M. (2014). Adult brain cancer in the U.S. black population: A Surveillance, Epidemiology, and End Results (SEER) analysis of incidence, survival, and trends. Med. Sci. Monit. Int. Med. J. Exp. Clin. Res..

[14] Ostrom Q.T., Cote D.J., Ascha M., Kruchko C., Barnholtz-Sloan J.S. (2018). Adult Glioma Incidence and Survival by Race or Ethnicity in the United States From 2000 to 2014. JAMA Oncol..

[15] Barnholtz-Sloan J.S., Maldonado J.L., Williams V.L., Curry W.T., Rodkey E.A., Barker F.G., Sloan A.E. (2007). Racial/ethnic differences in survival among elderly patients with a primary glioblastoma. J. Neurooncol..

[16] Barnholtz-Sloan J.S., Sloan A.E., Schwartz A.G. (2003). Racial differences in survival after diagnosis with primary malignant brain tumor. Cancer.

[17] Ostrom Q.T., Gittleman H., Fulop J., Liu M., Blanda R., Kromer C., Wolinsky Y., Kruchko C., Barnholtz-Sloan J.S. (2015). CBTRUS Statistical Report: Primary Brain and Central Nervous System Tumors Diagnosed in the United States in 2008–2012. Neuro-Oncology.

[18] McCormack R.M., Zhu P., Dono A., Takayasu T., Bhatia A., Blanco A.I., Tandon N., Ostrom Q.T., Gonzales A., Moreno S. (2021). Role of Ethnicity and Geographic Location on Glioblastoma IDH1/IDH2 Mutations. World Neurosurg..

[19] The King’s Fund The Health of People from Ethnic Minority Groups in England. https://www.kingsfund.org.uk/publications/health-people-ethnic-minority-groups-england.

[20] Braganza M.Z., Kitahara C.M., Berrington de González A., Inskip P.D., Johnson K.J., Rajaraman P. (2012). Ionizing radiation and the risk of brain and central nervous system tumors: A systematic review. Neuro-Oncology.

[21] Jack R.H., Davies E.A., Møller H. (2011). Lung cancer incidence and survival in different ethnic groups in South East England. Br. J. Cancer.

[22] Walter F.M., Penfold C., Joannides A., Saji S., Johnson M., Watts C., Brodbelt A., Jenkinson M.D., Price S.J., Hamilton W. (2019). Missed opportunities for diagnosing brain tumours in primary care: A qualitative study of patient experiences. Br. J. Gen. Pract..

[23] Lyratzopoulos G., Neal R.D., Barbiere J.M., Rubin G.P., Abel G.A. (2012). Variation in number of general practitioner consultations before hospital referral for cancer: Findings from the 2010 National Cancer Patient Experience Survey in England. Lancet Oncol..

[24] Mendonca S.C., Abel G.A., Lyratzopoulos G. (2016). Pre-referral GP consultations in patients subsequently diagnosed with rarer cancers: A study of patient-reported data. Br. J. Gen. Pract..

[25] Abel G.A., Shelton J., Johnson S., Elliss-Brookes L., Lyratzopoulos G. (2015). Cancer-specific variation in emergency presentation by sex, age and deprivation across 27 common and rarer cancers. Br. J. Cancer.

[26] Stupp R., Mason W.P., van den Bent M.J., Weller M., Fisher B., Taphoorn M.J., Belanger K., Brandes A.A., Marosi C., Bogdahn U. (2005). Radiotherapy plus concomitant and adjuvant temozolomide for glioblastoma. N. Engl. J. Med..

[27] Stupp R., Hegi M.E., Mason W.P., van den Bent M.J., Taphoorn M.J., Janzer R.C., Ludwin S.K., Allgeier A., Fisher B., Belanger K. (2009). Effects of radiotherapy with concomitant and adjuvant temozolomide versus radiotherapy alone on survival in glioblastoma in a randomised phase III study: 5-year analysis of the EORTC-NCIC trial. Lancet Oncol..

[28] Johnson K.J., Scheurer M.E., Woehrer A., Wiemels J. (2016). Evolving evidence on tumor and germline genetic classification of gliomas: Implications for etiology and survival studies. Clin. Neuropathol..

[29] Ohgaki H., Kleihues P. (2007). Genetic pathways to primary and secondary glioblastoma. Am. J. Pathol..

[30] Horvath S. (2013). DNA methylation age of human tissues and cell types. Genome Biol..

[31] Crimmins E.M., Thyagarajan B., Levine M.E., Weir D.R., Faul J. (2021). Associations of Age, Sex, Race/Ethnicity, and Education With 13 Epigenetic Clocks in a Nationally Representative U.S. Sample: The Health and Retirement Study. J. Gerontol. Ser. A.

[32] Kitahara C.M., Linet M.S., Brenner A.V., Wang S.S., Melin B.S., Wang Z., Inskip P.D., Freeman L.E.B., Braganza M.Z., Carreón T. (2014). Personal history of diabetes, genetic susceptibility to diabetes, and risk of brain glioma: A pooled analysis of observational studies. Cancer Epidemiol. Biomark. Prev..

[33] Zhao L., Zheng Z., Huang P. (2016). Diabetes mellitus and the risk of glioma: A meta-analysis. Oncotarget.

[34] Whyte M.B., Hinton W., McGovern A., van Vlymen J., Ferreira F., Calderara S., Mount J., Munro N., de Lusignan S. (2019). Disparities in glycaemic control, monitoring, and treatment of type 2 diabetes in England: A retrospective cohort analysis. PLoS Med..

[35] Klinger N.V., Mittal S. (2016). Therapeutic Potential of Curcumin for the Treatment of Brain Tumors. Oxidative Med. Cell. Longev..

[36] Das B.R., Tangri R., Ahmad F., Roy A., Patole K. (2013). Molecular investigation of isocitrate dehydrogenase gene (IDH) mutations in gliomas: First report of IDH2 mutations in Indian patients. Asian Pac. J. Cancer Prev..

[37] Dasgupta A., Gupta T., Jalali R. (2016). Indian data on central nervous tumors: A summary of published work. S. Asian J. Cancer.

[38] Deng L., Xiong P., Luo Y., Bu X., Qian S., Zhong W., Lv S. (2018). Association between IDH1/2 mutations and brain glioma grade. Oncol. Lett..

[39] Watanabe T., Nobusawa S., Kleihues P., Ohgaki H. (2009). IDH1 mutations are early events in the development of astrocytomas and oligodendrogliomas. Am. J. Pathol..

